# The Effects of Diet Formulation on the Yield, Proximate Composition, and Fatty Acid Profile of the Black Soldier Fly (*Hermetia illucens* L.) Prepupae Intended for Animal Feed

**DOI:** 10.3390/ani9040178

**Published:** 2019-04-19

**Authors:** Pier Paolo Danieli, Carola Lussiana, Laura Gasco, Andrea Amici, Bruno Ronchi

**Affiliations:** 1 Department of Agriculture and Forest Sciences, University of Tuscia, 01100 Viterbo, Italy; amici@unitus.it (A.A.); ronchi@unitus.it (B.R.); 2 Department of Agricultural Forest and Food Sciences, University of Torino, 10095 Grugliasco (TO), Italy; carola.lussiana@unito.it (C.L.); laura.gasco@unito.it (L.G.)

**Keywords:** low-value feedstocks, cereal byproducts, growth performance, diet optimization, insects for feed, fatty acid apparent concentration factor

## Abstract

**Simple Summary:**

Mass rearing of the black soldier fly to be used as feed is still at an early stage. Among the different issues, larval feeding and nutrition of this species are the most relevant ones from a practical standpoint. For example, testing four different diets, we found that this insect can be efficiently reared on wheat byproducts in place of cornmeal and that using diets richer in carbohydrates or proteins can negatively affect protein accumulation, larval development, and survivorship. Accumulation of unsaturated fats in black soldier fly prepupae is a matter of great interest, and it was found to be directly dependent on the amount of these fats in the rearing substrates. By appropriately mixing different food byproducts as diet ingredients, our research suggests that black soldier fly prepupae meal suitable for the feed formulation can be obtained.

**Abstract:**

The black soldier fly (BSF; *Hermetia illucens* L.) is a very promising insect species due to the ability to convert low-value substrates in highly nutrient feed. This work aimed to study the effect of three nominally isoenergetic diets containing plant ingredients such as barley, alfalfa, and wheat byproducts, formulated to be higher in nonfiber carbohydrates (TMD1), fibers (TMD2), and protein (TMD3) in comparison to an extensively genetic modified cereal (cornmeal)-based diet (C), on the growth, yield, and nutritive traits of BSF prepupae (BSFPs). Three growing trials with four biological replicates were carried out. Proximate and fatty acid analyses were performed on the diets and BSFPs. Feed conversion ratios (FCR), dry matter and nutrient yields, and apparent concentration factors (aBCF) for fatty acids (FAs) were calculated. Diet formulation had a substantial effect on the survival, development rate, and larval yield, but the FCR was unaffected. The BSFPs fed TMD3 did not result in a higher crude protein content in comparison to the C or TMD2 diets. Despite the leveled fat content of the diets, BSFPs reared on TMD1 were highest in fat, saturated FA, and fat yield. An apparent bioconcentration factor (aBCF) value lower than unity that was found for the unsaturated FA suggests that the BSFPs inefficiently absorb them from the diet or possibly turn them into saturated FA. However, the unsaturated FA accumulation in BSFPs depended on the levels that were found in the diet, which suggested some possibilities for the FA profile modulation. Overall, the TMD2 performed well despite the low-value of its main ingredients and high fiber content and can be considered to be a feasible option for the mass rearing of BSFPs that are intended for animal feed.

## 1. Introduction

The increased demand for products of animal origin requires an increase in feeding raw materials, and, to meet the needs of a market that is paying an increasing amount of attention to environmental issues, new sustainable raw materials are needed [1,2]. As a raw material, insects are a focus of one of the emerging frontiers of research, and publications indicate how they can suitably be used in animal feeds [3,4,5,6,7]. Insect-derived products have a chemical composition that meet the nutritional requirements of different animals well. They are rich in crude protein, essential amino acids, lipids containing fatty acids with anti-microbial properties (such as lauric acid), and micronutrients [8,9]. The composition is related to the species and can be modulated through the rearing substrate [10,11,12]. In addition to nutrients, a crucial aspect to consider for the suitability of an insect species for feed purposes is the possibility of being mass-reared to deliver large quantities to the market at an affordable price [13,14,15,16]. The mass rearing of insects requires the development of artificial diets that are able to meet nutritional requirements and to guarantee good insect-rearing performance. Several substrates have been tested, and some insect species are also considered to be valuable for waste management or the upgrading of low-value organic byproducts [17]. The black soldier fly (*Hermetia illucens* L., Diptera: Stratiomydae) has been indicated as a possible insect species and is one of the most appropriate [12,18]. However, it is important to underline that insects reared within the Europe Union are considered to be “farmed animals” (Reg. (EC) 1069/2009), and, therefore, several restrictions are applied to the allowed substrates. For instance, no catering waste, former foodstuffs containing meat or fish, or manure/animal feces are allowed. Black soldier fly (BSF) larvae (BSFLs) can be mass-reared on several organic substrates [11,12,18] and are rich in valuable nutrients. Moreover, trials indicate that they can be included in livestock and fish diets with promising results [5,19,20]. The BSFLs have been proven to grow easily (for example, see [21,22,23]) on diets developed for rearing house fly larvae (*Musca domestica* L.; Diptera: Muscidae) [24] that include alfalfa, wheat bran, and cornmeal. Notwithstanding, an in-depth evaluation of the suitability of artificial diets to fulfill the nutritional requirements of BSFLs (e.g., energy density, protein content, and aminoacidic or mineral profiles) has not been carried out. However, indirect evidence suggests that a balance between the crude protein and carbohydrate concentrations of the growing substrates may play a role in determining the optimal growth and compositional traits of BSFLs [12,18,25]. A formulation of diets for BSFLs comprising cornmeal, a feedstuff coming from *Zea mays* L., which is the second most-cultivated GM-plant in the world [26], implies that transgenic material can enter the BSFL production chain easily. In contrast, cereals such as spring wheat (*Triticum aestivum* L.) and barley (*Hordeum vulgare* L.) are cultivated worldwide and, in EU in particular, are not GM; thus, substituting corn with wheat or barley and/or their byproducts can ensure that no GM materials enter the BSFL production chain, provided that the diet does not include other potentially GM feedstocks (e.g., soybean, cotton seeds, canola, and their derivatives). If other rules for organic production are respected, the obtained BSFLs can be used in animal feed (e.g., in poultry feed) managed under organic farming principles. Therefore, the main aim of this research was to study the growing performance, yields, proximate composition, and fatty acid profile of BSF prepupa (BSFP) meal when larvae were fed on a control diet that includes cornmeal and three iso-energetic experimental diets that were formulated by using barley and low-value wheat-derived feedstuffs, with each differing in nonfiber carbohydrate, fiber, and crude protein concentrations.

## 2. Materials and Methods

All the BSF rearing and analytical procedures described hereafter were carried out at the Minilivestock Facility of the Experimental Farm “N. Lupori” (Viterbo, Italy) and in the Animal and Production Science laboratories of the University of Tuscia - DAFNE (Viterbo, Italy) and the University of Torino-DISAFA (Torino, Italy). Throughout the whole experiment, the BSFLs were obtained from three consecutive generations of flies (3rd–5th), starting with a single prepupa pool, which were provided by the University of Torino (Italy).

### 2.1. Black Soldier Fly Rearing

#### 2.1.1. Production of BSF Breeders, Mating and Oviposition

Pupation (200–300 pupae per batch) was performed in wood shavings according to Holmes et al. [27] and after the emergence, adult BSFs were maintained in an 80 × 35 × 85 cm flight cage made of a semi-rigid, self-supporting steel net (3 × 3 mm mesh) covered by a 3 mm thick methacrylate top. To ensure a high mating and deposition rate [28], four F36W/840 cool white neon tubes (Feilo Sylvania GmbH, Erlangen, Germany) and four 10 W LED light spots (Bion LIGHTING, Canton, China) were installed 10 cm above the flight cage in an assembly scheme that is similar to that of Nakamura et al. [29]. The lighting system ensured a total photonic flux of 1097 µmol/m^2^/s at the center of the flight space (total irradiance 52.7 W/m^2^, total illuminance 6.12 Klux; data recorded using an SS1 SunScan, Delta-T Devices Ltd., Cambridge, UK). Tap water was provided ad libitum in shallow plastic plates. Some branches of plastic climbing plants 70 cm long were available inside the cage to allow for the free expression of the flies’ lekking behavior [30]. Mating and oviposition took place at 28.1 ± 0.3 °C and 78.9 ± 5.3% RH. Mated females laid clutches of eggs within an oviposition box [29], in which packs of single-face fluted cardboard strips (approximately 3 flutes/cm) were suspended approximately 3 cm above a thick layer of wheat middlings in water (ratio of approximately 1:2) as an attractant.

#### 2.1.2. Production of BSFLs

Every day at 9:00 a.m., the oviposition packs were opened, and the cardboard strips were checked for clutches; if present, the clutches were transferred to thin plastic strips (six per strip) with the aid of a small steel rounded-end spatula. Plastic strips were then suspended at approximately 1 cm above a layer of 15 g wheat middling saturated with water (an approximate ratio of 1:1) into small circular starter larvaria (12 cm ID, 8 cm high). For the first 2–3 days, the starter larvaria were closed with a lid to avoid possible drying of the eggs, and, after hatching, finely holed lids were used after hatching. Three days after hatching, young BSFLs were fed with a double amount (30 g) of soaked wheat middlings and were divided into two parts if necessary. At six days from hatching, a part of the young BSFLs was used for the experimental purposes (see Section 2.3), and the rest was reared at a density of 10,000 larvae/m^2^ on the cornmeal/alfalfa/wheat bran diet (50:30:20; feeding rate 12.2 mg/day) to obtain a successive cohort of breeders.

### 2.2. Experimental Larvae Preparation, Seeding, and Harvesting

The six-day old BSFLs were separated from growing substrates by washing with tap water and then were classified dimensionally by a sieving system that was obtained by piling up two stainless steel ASTM test sieves (Giuliani Tecnologie s.r.l., Torino, Italy). A bunch of larvae was spread on the overlying 2 mm (10 mesh ASTM E11-70) sieve and was left to actively pass through it and the 1 mm sieve below (18 mesh). To make the process faster, the upper sieve was exposed to the direct light of a cool-white low-energy lamp (10 W). The BSFLs were frequently sprayed with tap water to prevent drying. Only the BSFLs retained by the 1 mm sieve were used in the growing trials. Triplicate seedings of ninety BSFLs were performed in 9.5 × 6.4 × 3.8 cm plastic larvaria at a density of 1.5 BSFLs/cm^2^. Before and after seeding, all the larvaria were individually weighed with a PE 160 analytical scale (Mettler Toledo S.p.A., Novate Milanese, Italy) to measure the total and mean larval weights. Each larvarium had a lid with a central hole (3 cm D) covered with a nylon net to ensure fair gaseous exchanges but was sufficiently fine to prevent the escape of BSFLs. Just after the seeding and weighing operations, the right amount of diet and water for the first 3-day feeding period was dispensed in all experimental larvaria. The BSFLs were reared until approximately 70% of them reached the prepupal stage (dark brown colored) [29]. Previous preparatory trials suggested that a 21-day growing period, as described for the experimental conditions that are detailed elsewhere in this section, would have been fit for this purpose.

### 2.3. Diets and Feeding

The whole experiment included three consecutive trials lasting 21 days each. For each trial, three separate batches of six-day old BSFLs were reared with four different diets: the control diet (C) and three experimental total mixed diets (namely, TMD1, TMD2, and TMD3) differing as far as the formulation was concerned (Table 1). The control diet was used for more than six months before the experimentation to rear BSFLs for other research purposes. The TMD1 and TMD2 were formulated to be nominally isoenergetic and isoproteic according to the gross energy (GE) and crude protein (CP) concentrations that were calculated for the C diet (estimates not shown) by using the nutritive data sheets that are available in the literature [31,32]. The adopted formulation made the TMD1 and TMD2, nominally, a high nonfiber content diet and a high fiber content diet, respectively (estimates not shown).

### 2.4. BSF Larvae Harvesting and Processing

At the end of the growing time (at 9:00 a.m. of the 22nd day after seeding), all the larvaria were weighed and immediately frozen at −18 °C. After at least 24 h, the larvaria were left at room temperature until complete defrosting. The BSFLs were then mechanically separated from the undigested diets and feces (frass) and were thoroughly washed with tap water. After washing, the BSFLs were dried over several layers of adsorbent laboratory paper. This procedure was carried out several times until no visible moisture was seen. For each experimental larvarium, the BSFLs and postfeeding larvae (or prepupae, BSFPs) were separately counted and weighed. Both BSFLs and BSFPs were then dried in an air-forced oven at 45 °C until a constant weight to determine the dry matter (DM_45_) and were finely ground with a high-speed bench mill. Larval meal samples were then stocked for further laboratory operations. Due to the low amount of recovered dry larval material, for each treatment, it was decided to pool the content of two larvaria that were randomly chosen within the four replicates that were set up. In some diets, the recovered dry BSFL meal was very low, and only the BSFP meal samples were further submitted to proximate and chemical evaluation. In comparison to the last feeding larval stages (i.e., the 6th instar), prepupae might offer two advantages [18]: (i) the prepupa empties its digestive tract, thereby reducing the risk of carrying pathogenic microorganisms; and (ii) the prepupal migrating behavior offers opportunities for harvesting in a scaled-up rearing system. After the BSFP separation, samples of frass were diluted in ultrapure water (ASTM type I standard, specific conductivity 0.055 µS/cm) in a ratio of 1:3, and the pH was measured using a portable pH-meter (XS Instruments, Carpi, Italy) calibrated in the range 4.01–10.01.

### 2.5. Proximate Composition of Diets and BSFP Meal

#### 2.5.1. Diets

The proximate composition and gross energy (GE) content were determined for the control diet and for the three experimental diets. All the analytical determinations were performed twice. Gravimetric measurements were carried out by using a PE160 analytical scale (Mettler Toledo S.p.A., Novate Milanese, Italy). The dry matter content (DM) of the diet samples was determined by gravimetry (AOAC method n. 934.01) [33]. Gravimetric determination of the ash content (ASH) was obtained after sample incineration by the muffle furnace (AOAC method n. 942.05) [33]. Crude protein (CP) was assessed through the Kjeldahl method (AOAC method n. 978.04) [33], and the ether extract (EE) was determined by Soxhlet extraction (AOAC method n. 920.39) [33]. Two fibrous fractions, the neutral detergent fiber (NDF) and the acid detergent fiber (ADF), were also analyzed. The neutral detergent fiber was determined by boiling a 0.5 g sample for 1 h in 100 mL of neutral detergent plus 0.05 mL of heat-stable α-amylase (ANKOM Technology, NY, USA) and 0.5 g of analytical grade sodium sulfite (Sigma-Aldrich, Darmstadt, Germany) [34]. The acid detergent fiber was determined as described by Goering and Van Soest [35]. All compositional data were measured in g/kg DM. The GE content (MJ/kg DM) of the diet samples was obtained through a Parr 6200 Isoperibol Calorimeter (Parr Instrument Company, Moline, IL, USA) according to the manufacturer’s instructions. The pH of the diets was determined in subsamples diluted with ASTM type I water as was detailed for frass (see Section 2.4).

#### 2.5.2. BSFP Meal

The concentrations of DM at 105 °C, CP, EE, and ash were obtained by using the corresponding methods as were previously described for the diets. In addition, the chitin content of the BSFP meal was analyzed according to Liu et al. [36], with minor modifications. Briefly, an aliquot of the prepupae meal (90–100 mg) was sealed in an ANKOM filter bag (ANKOM Technology, Macedon, NY, USA) that was shaped to fit a 15 mL screw cap centrifuge tube and was submitted to demineralization for 30 min in 5 mL of HCl 1 M at 100 °C. The five washing steps in ASTM Type I water that were completed to reach neutrality followed the demineralization process. A deproteinization step was then performed in 5 mL NaOH 1 M at 80 °C for 24 h. In the end, the sample was washed five times in ASTM Type I water until neutrality was reached. After drying at 105 °C in an air-forced oven for 2 h, the chitin content (CT, g/kg DM) was calculated as follows:

(1) $$CT=1000\times \;\frac{Fw-\left(Bw\times C\right)}{Sw}$$

where *Fw* = weight after demineralization, deproteinization, and drying (g), *Bw* = weight of the modified ANKOM extraction bag (g), *C* = dimensionless factor taking in account the mean weight loss of extraction bags (0.999, n = 6) treated according to the same procedure used for the samples, and *Sw* = exact amount of sample processed (g). The discoloration step proposed by Liu et al. [36], by soaking samples in 1% KMnO_4_ for 1 h was omitted because propaedeutic trials gave no improvements in terms of weight loss of the demineralized-deproteinized samples after treatment with the permanganate solution (Table S1, supplementary materials).

### 2.6. Fatty Acid Profiles of Diets and BSFPs

Representative samples of dried (at a temperature of 45 °C) rearing substrates and BSFP meal were analyzed for the fatty acid (FA) composition as described by Alves et al. [37]. Briefly, FAs were extracted and directly trans-esterified as described by Sukhija and Palmquist [38]. Solid-phase extraction (SPE) with LiChrolut Si cartridges (Merck KGaA, Darmstadt, Germany) was used as a cleanup method to remove interfering compounds. Fatty acid methyl esters (FAMEs) were separated and quantified using the same analytical instruments and temperature program as described by Renna et al. [39]. Peaks were identified by injecting pure FAME standards (Sigma–Aldrich, Milano, Italy; Matreya Inc., State College, PA, USA; Restek Corporation, PA, Bellefonte, USA) and by comparison with the chromatogram published by Alves et al. [37]. Quantification was assessed using heptadecanoic acid as the internal standard. The results were expressed as mg/100 g DM and were reported as g/100 g of the total detected fatty acids (TDFAs)

### 2.7. Data Elaboration and Statistical Analysis

On the data regarding BSFPs and BSFLs recorded at the end of each trial, the ratio of BSFPs to total BSF specimens recovered was calculated. The survival rate (SR) was also calculated by dividing the total BSF specimen recorded at harvest by the number of larvae at the beginning of each trial (90). The feed conversion ratios (FCRs) based on the wet weight (FCR_W_) and dry matter (FCR_D_) were calculated as follows:

(2) $$FCR_{W}=TD_{WW}/TWG$$

(3) $$FCR_{D}=TD_{DW}/TWG$$

where *TD_WW_* = total weight of diet administered to each larvarium in 21 days (g), *TD_DW_* = total dry weight of diet administered to each larvarium in 21 days (g), and *TWG* = total weight gain of BSF specimens regardless the developments stage (final weight minus initial BSFLs weight) at harvest (g). For all the above parameters, four replicates per treatment within a trial were considered in the statistical analysis. For the BSFP meal, from the proximate composition (see Section 2.5.2), the CP content of the defatted dry matter (CP_DEFATTED_) (g/kg DM) was calculated as follows:

(4) $$CP_{\mathrm{DEFATTED}}=CP\times \frac{1000}{\left(1000-EE\right)}.$$

The nonfiber carbohydrates content (NFC) (g/kg DM) was estimated as follows [40]:

(5) NFC=1000−(CP+EE+ASH+NDF).

Similarly, the residual organic matter (ROM) (g/kg DM) was estimated by calculation from the proximate compositional data on BSFPs:

(6) ROM=1000−(CP+EE+ASH+CT).

The dry matter, crude protein, ether extract, and ROM yields (DMY, CPY, EEY, and ROMY, respectively) were calculated from the DM, CP, EE, and ROM concentrations of the harvested BSFPs and the relative total weight (wet or dry) values. As far as the fatty acid groups are concerned, the total saturated, unsaturated, monounsaturated, monounsaturated *trans*, polyunsaturated, and branched FAs (ΣSFA, ΣUFA, ΣMUFA, ΣMUFA *trans*, ΣPUFA, and Σiso + anteiso FA) were obtained by summing up all the relative FA detected, identified, and thus quantified. In addition, the ratios ΣUFA/ΣSFA, ΣMUFA/ΣSFA, ΣPUFA/ΣSFA, and ΣPUFA/ΣMUFA were also calculated for the unprocessed point data. For each FA or FA group, an apparent bioconcentration factor (aBCF) was calculated as follows:

(7) $$aBCF_{i}=\frac{{\left(FA_{i}/TDFA\right)}_{BSFP}}{{\left(FA_{i}/TDFA\right)}_{DIET}}$$

where *i* = specific FA (g/100 g DM) or the sum of a group of FA (SFA, MUFA, MUFA *trans*, PUFA, and branched FA), and *TDFA* = total detected fatty acids (g/100 g DM).

All the data were analyzed by ANOVA through the General Linear Model procedure of Statistica 10 (StatSoft Inc., Tulsa, OK, USA) to detect the diet effect according to the model:

(8) $$y_{ij}=\mu +D_{i}+\varepsilon_{ij}$$

where *y* = the single datum, *µ* = the overall mean, *D* = diet (*i* = 4, C, TMD1, TMD2, and TMD3), and *ε* = the error term. The difference between means was tested through Fisher’s least significant difference (LSD) post-hoc statistic (StatSoft Inc., Tulsa, OK, USA). Significance was declared at *p* ≤ 0.05. Except for the proximate composition data and fatty acid profile of the diets, all the results are presented as the mean ± standard error.

## 3. Results

### 3.1. Proximate Composition and Fatty Acid Profile of the Diets

The chemical composition of the control and experimental diets is reported in Table 2. The CP concentrations of TMD1 and TMD2 were within a ± 5% range of that of the control diet. The TMD3 was slightly higher than CP excess designed during the experimental planning stage (+30%). The EE content was fairly leveled among diets. In contrast, TMD1 and TMD2 resulted in a lower (−38.7%) and a higher (+31.5%) fibrous carbohydrate content (NDF), respectively, compared to that of the control. Similarly, the content of lingo-cellulosic fiber fraction (ADF) was higher in TMD2 and lower in TMD1 than in C because of the different formulations (Table 1). Accordingly, the NFC content varied from −14.4% for TMD2 up to +15.4% for TMD1 in comparison to that of the control diet. The GE values showed low variations, as expected.

The fatty acid profiles of the diets are listed in Table 3. Depending on the diet formulation, the total percentage of SFA ranged from 27.3 (C) to 35.1 (TMD1). Overall, in comparison to the FA profile of the control diet, the lower content of MUFA was counterbalanced by a higher content of SFA in TMD1 or PUFA in TMD2 and TMD3. As regards the pH of the wet diets, which was measured at the beginning of the first trial, the recorded values were 5.95, 5.60, 5.90, and 5.79 for C, TMD1, TMD2, and TMD3, respectively.

### 3.2. BSFP Recovery, Larval Survivorship, and Yields

The diet had a highly significant effect on the survival and development of BSFLs in our rearing conditions (Table 4). In comparison with the control diet, the TMD1 exhibited a reduction of BSFPs and total BSF counts at harvest and a lower survival rate. Lower total BSF counts and survival rate was also observed for the TMD3 if compared to the control diet. As far as the prepupae to the total BSFP count at harvest, TMD1 gave also worse results than TMD2 (*p* < 0.01) and the protein-rich TMD3 (*p* < 0.05). On the other hand, TMD3 did not give better results concerning TMD2 or diet C.

At harvest, the weight of the BSFPs that were reared on experimental diets TMD1–TMD3 did not differ from those that were reared on the C diet, but a higher weight (+39.6%) was observed for the BSFPs reared on the TMD2 if compared to the TMD1 counterparts. However, total yield was affected by the diet formulation with significant differences recorded for TMD3 BSFs against the C diet (*p* < 0.01) and TMD1 (*p* < 0.05). Neither the feed conversion ratio calculated on the total weight of diets as fed (FCR_W_) nor that calculated on the diet DM basis (FCR_D_) was affected by diet formulation. Diet formulation had a stronger effect on the survival and development rate of BSFLs than on the yield in terms of larvae live body weight, except the high-protein diet (TMD3) for which an unexpected low overall yield was observed.

### 3.3. BSFP Proximate Composition and Nutrient Yields

Among the three experimental diets, only the BSFPs reared on TMD1 showed a significant difference compared with controls (Table 5). Notably, the BSFPs reared on TMD1 exhibited a lower CP, CP_DEFATTED_, and ROM content (−34.7%, −18.3%, and −14.4%, respectively) than controls; it did, however, have a higher fat content (+42.1%). The high-protein diet (TMD3) did not result in any practical advantage (e.g., a significant higher CP content) in comparison to the isoenergetic control diet or TMD2. No significant difference was observed between control and experimental diets as far as the chitin and ash concentrations of BSFPs.

Combining biomass and proximate data, the yields of DM, CP, EE, and ROM were estimated (Table 6). According to the high EE content of BSFPs reared on TMD1, the related EEY value was higher (*p* < 0.01) than the BSFPs reared on the control diet and on the high-fiber TMD2 and high-protein TMD3. No significant differences were recorded as far as the DM, ROM, and crude protein yields per larvarium.

### 3.4. Fatty Acid Profiles of BSFPs and the Apparent Bioconcentration Factors

The FA profiles of the BSFP meal were affected by the diets that the larvae were reared on (Table 7). The SFA group was overrepresented (*p* < 0.01) in TMD1 prepupae in comparison with both the control (+9.2%) and the other experimental diets. For the three highly represented SFA, the lauric acid content (C12:0, overall mean 54.31 g/100 g of TDFA) and myristic acid content (C14:0, 8.77 g/100 g of TDFA) were higher in TMD1 than in the control, and the TMD2–TMD3 prepupae, except palmitic acid (C16:0, 12.04 g/100 g of TDFA), did not differ among the BSFPs reared on different diets. In contrast, the stearic (C18:0) and arachidic (C20:0) acids showed an opposite pattern to the shortest chain SFA, with the values recorded in the prepupae that were reared on TMD1 being lower than those that were reared on the control diet (C18:0) or on TMD2–TMD3 (C20:0).

**Figure 1:**
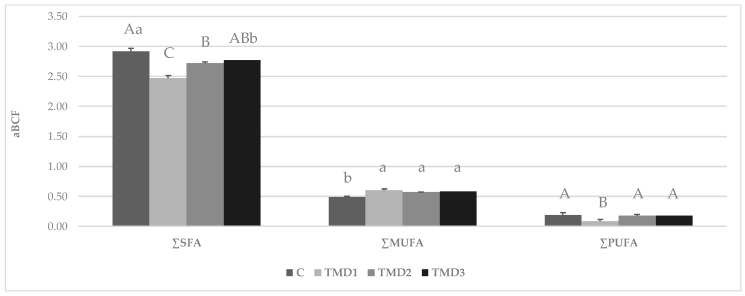
Effect of the diet on the apparent bioconcentration factors for the total identified saturated fatty acids (SFA), monounsaturated fatty acids (MUFA), and polyunsaturated fatty acids (PUFA) in the control (C) and experimental diets (TMD1: high-nonfiber carbohydrate content total mixed diet; TMD2: high-fiber content total mixed diet; TMD3: high-protein content total mixed diet). ^A,B,C^ capital letters above the means indicate a significant difference at *p* < 0.01; ^a,b^ letters above means indicate a significant difference at *p* < 0.05.

On average, the MUFA accounted for 9.2% of all the TDFA, but significant differences were observed among the diets. The BSFPs reared on TMD1 had an MUFA content, especially the oleic acid (C18:1 *cis* 9), that was lower than the those on C, TMD2, or TMD3. Among all the identified FA, PUFA accounted for as much as 8.6%, with differences among the diets that are similar to those recorded for MUFA. Mainly, TMD1 prepupae were approximately half lower in MFA and PUFA than in the experimental counterparts, with considerable differences in the linoleic (C18:2 n-6) and α-linolenic (C18:3 n-3) acids. All the ratios presented in Table 6, including the total identified SFA, UFA, MUFA, and PUFA, resulted in lower values for BSFPs reared on TMD1 (*p* < 0.01) in comparison with those reared on C, TMD2, and TMD3. The degree to which each detected and quantified FA in the diets tended to accumulate in BSFP fat matter was estimated by the calculation of specific apparent bioconcentration factors (Table 8 and Figure 1). According to the method of calculation proposed here (see Equation (6)), the aBCF values higher than unity, as for many SFA and for palmitoleic acid (C16:1), have to be regarded as indices of apparent accumulation in the BSFPs concerning the levels found in the diets. Except for the oleate (C18:1 *cis* 9), the diet had a substantial effect on all the calculated aBCF values.

Considering the main groups of FA (Figure 1), the highest aBCF values were recorded for the total SFA. For this FA group, the aBCF values were higher than unity regardless of the diet. However, the BSFPs reared on the control diet tended to accumulate more saturated FA than BSFs reared on the isoenergetic TMD2 and TMD3. In contrast, BSFPs reared on TMD1 exhibited the lowest aBCF for SFA, even if that diet was richer in SFA than were the others (see Table 3). Overall, the aBCF of the unsaturated FA were lower than unity, which indicated that BSFPs did not absorb unsaturated FA from diets or possibly turned ingested UFA into more saturated FA. Some significant differences among the diets were recorded with BSFs reared on the C diet, which showed a lower tendency (*p* < 0.05) to accumulate MUFA and that the TMD1-reared BSFs showed the lowest (*p* < 0.01) aBCF value for PUFA.

Overall, the saturated FA in BSFPs was poorly correlated with the levels that were found in their diets, with the exception of myristate (C14:0) and palmitate (C16:0), for which positive and negative correlation coefficients were observed, respectively. As regards the unsaturated FA, with the exception of linoleic acid, all significant correlations between the specific FA in the diet and the respective levels found in BSFPs were positive, which suggested that there is a general tendency for BSFPs to reflect the differences of these FA among the diets (Table 9). Branched-chain FA in BSFPs was negatively correlated with the levels that were found in their diets.

## 4. Discussion

BSFLs are generalist detritivores [25] that are able to efficiently convert a wide range of organic substrates into high-protein larval biomass suitable for farm animal feeding purposes [41,42]. Despite the practical demonstration of using BSF larvae for poultry and pigs [14] as well as farmed fishes [43], feeding optimized, low-value diets for rearing on this insect have not been proposed until now. More importantly, it seems that the nutritional requirements of BSFLs are not well investigated [10], and a complete understanding of the nutrition physiology of this insect species is far from being achieved. However, for successful mass rearing of BSFs, the high standard of conversion efficiency, substrate reduction, and nutritive principles accumulation in a larval meal cannot be reached if the nutrition requirements of this species are not well understood. In other words, if BSFs or other insects are to become a more mainstream food or feed source, diets must be optimized to allow for the cheapest mass production [15]. Of the four diets tested in this research, the high NFC and high CP concentrations in TMD1 and TMD3 led to the worst results in terms of larval survivorship, but TMD1 also yielded poor results for the BSFP to total BSFL ratio and the total BSFP weight (Table 4). The role of non–fiber carbohydrates is poorly studied in insects, particularly in dipteran species. However, it seems that dipteran larvae can survive and grow with zero- or low-carbohydrate diets due to their ability to obtain the energy for maintenance and body weight gain from proteins [44]. In contrast, an excess of starch and sugars has been shown to exert adverse effects on the growth performance of the house fly reared under strictly controlled conditions [44]. Recently, it has been shown that the protein-to-digestible-carbohydrate ratio plays a significant role in the select life-history traits of BSFs, even though its effect may be widely modulated by the moisture content of diets [25]. In particular, excess carbohydrates (35% DM, CP = 7% DM, 70% diet moisture as fed) caused a significant delay (0.4–6.9 days) in the time required for 40% of the BSFLs to become prepupae in comparison with a more balanced diet [25]. In our experimental condition, the low BSFP/total BSFL ratio recorded for TMD1 (Table 4) can be interpreted as a result of the delayed passage from feeding larvae to prepupae, as was described in other studies [23,25] and is imputable to the high NFC content of that diet in comparison with the others under experimentation. The TMD1 also gave worst results for the total BSFP weight at harvest but performed better than the high-protein TMD3, if the total BSF (feeding larvae and prepupae) weight is considered. For the high CP content of BSFL diets, Tschirner and Simon [10] observed a low survival rate of BSFLs reared on a high-protein substrate (dried distillers with solubles, CP = 312 g/kg DM) in comparison with other BSFLs fed on a control diet having 238 g/kg DM crude proteins (SR = 21.7% and SR = 55.6%, respectively). Compared to those findings, the overall survival rates observed in our study were higher, but differences between high-protein diet TMD3 and C or TMD2 penalized the survivorship of growing BSFLs. In addition, the high-protein TMD3 diet yielded a lower BSF weight at harvest compared with TMD1, which suggested that, under our experimental conditions, having a high-protein content in the diet of BSFs exerted a more significant impact than did an excess of digestible carbohydrates. This observation might be explained by the energetic costs that are related to the detoxification of proteinogenic nitrogen excess. In partial support of this hypothesis, it has been shown that the metabolic rate at 27 °C of BSFLs reared on a protein-rich diet is higher than that of BSFLs reared on more balanced diets [23]. Furthermore, high-protein diets have detrimental effects on the survivorship and growth of the honey bee (*Apis mellifera* L.; Hymenoptera: Apidae) [45] and cotton bollworm (*Helicoverpa zea* Boddie; Lepidoptera: Noctuidae) [46] larvae. Under our experimental conditions, the final pH values recorded in TMD3 frass (8.77 ± 0.06) were significantly higher (*p* < 0.01) than in the other larvaria (7.25 ± 0.09 for C, 6.32 ± 0.05 for TMD1 and 8.12 ± 0.05 for TMD2), which suggested that a lower larval retention of nitrogen probably took place, as was hypothesized by Tschirner and Simon [10] about BSFLs reared on a high-protein substrate. It has to be underlined that TMD3 was not the diet with the highest pH value at the beginning of the trials (see Section 3.1). The high pH of the substrate obtained through manipulation by the addition of sodium hydroxide does not seem to be a problem for the development of BSFs [47], but it is also possible that the high pH value that was recorded in TMD3 frass during the harvest was an indicator of an excessive release of alkaline and toxic catabolites, such as ammonia [10].

The proximate composition of BSFPs is of prime importance in the view of their possible use as animal feed and in other applicative usages (e.g., biodiesel production). Among the four diets tested, the more protein-rich TMD3 did not yield any practical advantage, compared with the control diet or the high-fiber TMD2 (Table 5). In fact, the CP content of BSFPs reared on TMD3 was not different from the levels found in the C and TMD2 counterparts. These surprising results corroborate our observations about the low-growth performance shown by BSFLs reared on TMD3 but are in contrast with findings by other studies. For example, Tschirner and Simon [10] found that BSFLs reared on a proteinaceous substrate (CP = 31.2% DM) had a CP content of 44.6 ± 0.6% DM that was intermediate between their control (37.2 ± 0.7% DM) (middlings as a substrate, CP = 22.0% DM) and the fiber BSFLs (52.3 ± 1.7% DM) (sugar beet pulp as a substrate, CP = 8.5% DM). However, the data that were obtained on the larval pool (approximately 70% of BSFPs) on which those researchers carried out the laboratory analysis can only be approximately compared with our data that were obtained exclusively on BSFPs. Working on BSFPs, Tinder [48] found that mixing sorghum (CP = 3.5%) in different ratios with cowpea (CP = 7.7%) from 100 to 0% resulted in an increasing content of larval CP. Our diets, however, had a higher CP content (Table 2) when compared with the ones used by Tinder [48], and this may be a critical difference in the interpretation of the respective results. In another study [49], BSFLs were reared on different artificial diets formulated to have proteins (P) and fats (F) at high (H) or low (L) levels, which were combined in a 2 × 2 experimental design (HPHF, HPLF, LPHF, and LPLF). In these experimental conditions, it was shown that the CP content of BSFLs increased, passing from low- to high-protein substrates when the fat content was high (LPHF vs. HPHF), but there were no significant differences when comparing the BSFs reared on substrates with the lowest fat content (HPLF vs. LPLF) [49]. Thus, univocal relationships between the protein content in the BSF diet and the larval body are not always clear. In our study, despite the similarity in the fat content of the experimental diets (Table 2), the BSFPs reared on the high NFC diet (TMD1) were approximately 50% fatter than the others (Table 5). The higher amount of lipids that were accumulated in the body was, however, counterbalanced by a lower protein content. Because the gross energy content of TMD1 was similar to that of the C diet and TMD2, the main factor affecting the protein-to-fat content ratio in BSFPs might have been the protein-to-carbohydrate ratio of the diet. In fact, the TMD1 had an NFC content of 15 and 17% higher than C and TMD2, respectively, but had a similar CP content (Table 2). However, the causal mechanism underpinning this experimental evidence is not clear. Spranghers et al. [18] did not find significant differences between the fat levels in BSFPs reared on restaurant waste (NFC = 618 g/kg DM; CP = 157 g/kg DM) in comparison with control BSFPs reared on chicken feed (NFC = 425 g/kg DM; CP = 175 g/kg DM) or vegetable waste (NFC = 449 g/kg DM; CP = 86 g/kg DM). However, their experiments did not last a prefixed time, as in our study (21 days), because harvesting took place six days after the first prepupa appeared in each experimental treatment [18]. The development rate of BSFPs largely depends on the physicochemical traits of the diets, in particular, the protein-to-carbohydrate ratio, as shown by our data and by others’ data [25]. However, all the life-cycle phases of BSFs are characterized by dynamic changes in the body nutrient concentrations [50]. As a matter of fact, it has been shown that early prepupating BSFLs (E-BSFPs, i.e., larvae prepupating within the first two days from the appearance of the first BSFPs) have 20% less crude lipids than ones that prepupate later (L-BSFPs) [50]. Apart from any other possible consideration concerning other differences between our study and that of Spranghers et al. [18] (e.g., the fiber or GE concentrations), the different harvesting procedures might have introduced confounding factors that do not make the respective results directly comparable. However, the increased fat content of BSFPs reared on TMD1 can be explained by the demonstration of Li et al. [51], who showed that adding simple sugars (especially glucose, the monomeric unit of starchy matter) to a standard diet improved fat accumulation in the growing BSFLs. In our study, numerical differences were found in the ash content of BSFPs, and these differences roughly reflect the pattern of the ash content of the tested diets. A significantly different mineral accumulation in BSF larvae has been found by Tschirner and Simon [10] regarding the amounts that are available through the diet, but the differences in ash concentrations of the three diets tested by those authors were greater than the ones that were used in the present study. The fatty acid profile of BSFPs intended for animal feed is a fundamental issue in light of obtaining animal food products with healthy levels of UFA and specific FA ratios (e.g., n-6/n-3 FA, PUFA/SFA). A fortiori, the issue is particularly relevant if the insects and their derived products are to be directly developed for human consumption. To some extent, it has been shown that the FA composition of BSFLs can be modulated dynamically depending on the particular assortment of FA in their diet [52,53] and the duration of specific feeding regimes [54]. Despite this, some lipidic components play a functional role in BSFs and cannot be modulated at will. It is well documented that the dipterans have typically high concentrations of palmitic (C16:0), palmitoleic (C16:1), and oleic (C18:1) acids [55]. However, it is also known that some dipteran species can survive with diets excluding these FAs [44], which indicates that they are able to produce these FAs from other sources to meet specific physiological requirements. In BSFPs, we found a huge quantity of lauric acid (C12:0) that amounted to more than 50% of the TDFA, despite the very low levels of this FA that were found in the diets. Barroso et al. [53] obtained BSF larvae that are rich in lauric acid (>30% of total FA), even though this FA occurred in the diets at levels that are comparable to those in our experiment (<1% of total FA). In another study [52], lauric acid was as much as 43.4% of the total FA in BSFLs but was undetectable in BSFPs or other six promising insect species for aqua-feed applications. In our study, there were no significant differences among the diets as far as the capric acid, but there was more C12:0 (62.8% of TDFA) in the BSFPs that were reared on the NFC-richer diet (TMD1). Similar differences were observed when comparing the myristic acid content of BSFPs reared on TMD1 to that of the others (Table 7). These differences made the TMD1 BSFPs higher in SFA content than the others. Oonincx et al. [49] were able to detect capric acid (C10:0) in a range between 0.7 and 1.3% of TFA and laurate from 18.9 to 50.7% of TFA in BSF larvae that were reared on five different diets. Unfortunately, the data that were reported by Oonincx et al. [49] added little useful information on the complex picture regarding the partition of energy and/or FA provided with the diet to the BSF larvae FA, because they did not report other diet proximate values than that of dry matter and CP. However, their data show that the highest lauric and myristic acids concentrations were found in BSF larvae that were fed with low-fat diets (TFA ranging from 1 to 3.5%), regardless of the level of CP. It would, therefore, seem that a low-fat diet might be a condition that allows the shorter SFA to accumulate in the BSFLs [49]. However, it cannot be the only condition, because our experiments have clearly shown that diets that are similarly low as far as the fatty components (such as the C diet and TMD1) yielded very different levels of FA C12:0 and C14:0 in BSFPs. In an interesting study, Spranghers et al. [18] obtained quite different C12:0 and C14:0 concentrations among the BSFPs that were reared on four different substrates. In particular, a comparison between the results obtained feeding BSFLs with digestate (NFC = 7 g/kg DM; EE = 62 g/kg DM; insoluble fiber = 381 g/kg DM), chicken feed (NFC = 425 g/kg DM; EE = 53 g/kg DM, insoluble fiber = 175 g/kg DM), or vegetable waste (NFC = 449 g/kg DM; EE = 21 g/kg DM, insoluble fiber = 331 g/kg DM) highlights that lauric and myristic acids occurred at lower levels in the BSFPs that were fed digestate (436.5 and 68.7 g/kg TFA) than the BSFPs that were fed chicken feed (573.5 and 73.4 g/kg TFA) or vegetable waste (608.9 and 94.8 g/kg TFA) [18]. The high accumulation of C12:0 and C14:0 in BSFPs that were reared on the high NFC diet TMD1 was counterbalanced by the low level of several longer chains of saturated and unsaturated FA if compared to the other diets. In fact, with minor exceptions, the MUFA and PUFA of BSFPs reared on TMD1 were lower in comparison with BSFPs reared on the C diet as well as on TMD2 and TMD3. However, the correlation study that was performed clearly indicated that UFA levels in BSFPs are positively correlated with the respective levels occurring in the diets. This outcome is in agreement with the results of Barroso et al. [54], who obtained BSFLs enriched in n-3 FA by using diets at increasing concentrations of these FA.

## 5. Conclusions

In conclusion, the three isoenergetic experimental total mixed diets formulated with barley and wheat byproducts that were tested in this study yielded satisfactory results in terms of the dry matter and protein yields in comparison with the control diet composed of cornmeal. However, differences in the diet formulation, such the content of non–fiber carbohydrates (TMD1 against the other diets) caused different tendencies of fat accumulation by BSFPs, especially fat rich in SFA, at the expense of proteins and other BSFP constituents. However, the tendency of BSFLs to slow down their development rate when fed on a diet high in carbohydrates needs further investigation for its practical implications (e.g., the accumulation of fat desired for uses other than animal feeding). In terms of the diets’ energetic density, the diet formulation with increased crude protein content (TMD3) did not yield any practical advantage in terms of the larval growth, yield, composition, or fatty acid profile. In contrast, the experimental formulation including wheat middlings, wheat straw, barley, and alfalfa meal (TMD2) performed well despite the low value of its main ingredients and its high-fiber content. The preliminary results reported here could be the basis of future studies dealing with a systematic evaluation of the role of macronutrients (carbohydrates, proteins, fat), fibers, and their relative balances in driving the BSF larval growth, yield, and nutritive value.

## Figures and Tables

**Table 1:** Formulation (g/100 g) of the control (C) and the experimental diets (TMD1–TMD3) for black soldier fly larvae (BSFLs).

Feedstuffs	C	TMD1	TMD2	TMD3
Ground corn	50	-	-	-
Ground barley	-	68	16	15
Wheat middlings	-	-	50	55
Wheat bran	20	20	-	-
Dehydrated alfalfa	30	12	10	30
Wheat straw	-	-	24	-

TMD1: high-nonfiber carbohydrate content total mixed diet; TMD2: high-fiber content total mixed diet; TMD3: high-protein content total mixed diet In the same way, the TMD3 was formulated to obtain a 30% higher CP content than that of the control diet. Larval feeding was performed to provide 12.2 mg/larvae/day of one of the three experimental TMDs or the control diet. Before mixing, all the ingredients were ground to pass a 1-mm screen by a hammer mill (Retsch GmbH, Germany). Just before feeding, deionized water was added to the mixed ingredients at variable rates (in milliliters per gram of diet as fed) according to the water-holding capacity of the diets: 1.70, 1.63, 1.82, and 1.73 for C, TMD1, TMD2, and TMD3, respectively. All the experimental larvaria were fed concurrently at 2- or 3-day intervals according to the same schedule that was adopted for all trials. A single stock maintained in freezing conditions (−18 °C) was prepared for each diet to be used throughout the experimental period. All the trials were carried out at 28.5 ± 0.3 °C and 75.6 ± 4.2 % RH.

**Table 2:** Proximate composition (g/kg DM; unless otherwise stated) and gross energy content (MJ/kg DM) of the control (C) and experimental diets (TMD1–TMD3).

Proximate Composition	C	TMD1	TMD2	TMD3
DM (g/Kg WW)	875	871	895	882
Ash	33	24	41	41
CP	106	111	112	138
EE	42	40	44	40
NDF	222	136	292	220
ADF	105	96	196	116
NFC	597	689	511	561
GE (MJ/Kg DM)	18.6	19.4	18.8	19.0

DM = dry matter; WW = wet weight; CP = crude protein; EE = ether extract; NDF = neutral detergent fiber; ADF = acid detergent fiber; NFC = non–fiber carbohydrate; GE = gross energy. TMD1: high-nonfiber carbohydrate content total mixed diet; TMD2: high-fiber content total mixed diet; TMD3: high-protein content total mixed diet.

**Table 3:** Fatty acid (FA) profile (g/100 g of TDFA) of the control (C) and experimental (TMD1–TMD3) diets.

Fatty Acids	C	TMD1	TMD2	TMD3
C12:0	0.64	0.37	0.22	0.17
C14:0	0.50	0.60	0.59	0.40
C16:0	24.41	32.31	27.45	27.36
C18:0	1.50	1.57	1.12	1.09
C20:0	nd	nd	0.14	0.09
C14:1 + C15	0.20	0.21	0.20	0.20
C16:1	0.33	0.20	0.30	0.30
C18:1 *cis* 9	20.49	13.04	13.77	13.48
C18:1 *cis* 11	0.70	0.54	0.97	0.96
C18:2 n-6	46.60	46.40	49.92	49.84
C18:3 n-6	nd	nd	0.08	0.06
C18:3 n-3	4.21	4.26	4.34	5.18
C20:1 *cis* 11	nd	0.16	0.29	0.27
ΣSFA	27.31	35.11	29.76	29.33
Σiso + anteiso FA	0.27	0.26	0.23	0.22
ΣMUFA	21.72	14.15	15.71	15.33
ΣMUFA *trans*	nd	nd	0.18	0.13
ΣPUFA	50.96	50.74	54.53	55.34

SFA = saturated fatty acids; MUFA = monounsaturated fatty acids; PUFA = polyunsaturated fatty acids; nd = not detected. TMD1: high-nonfiber carbohydrate content total mixed diet; TMD2: high-fiber content total mixed diet; TMD3: high-protein content total mixed diet.

**Table 4:** Effect of the diets on larval survival and development, black soldier fly prepupae (BSFPs), and total yields and feed conversion ratios (FCRs).

Parameter	C	TMD1	TMD2	TMD3	SEM	*p*
Initial BSFLs (n)	90	90	90	90	-	-
BSFPs (n)	48.09 ± 3.55 ^A^	32.45 ± 3.39 ^B^	56.09 ± 4.61 ^Aa^	43.27 ± 4.32 ^ABb^	2.329	0.002
Total BSFs (n)	75.45 ± 2.14 ^A^	59.36 ± 2.53 ^B^	74.00 ± 3.70 ^A^	60.73 ± 3.64 ^B^	1.862	0.001
BSFPs/Total BSF	0.64 ± 0.05 ^AB^	0.54 ± 0.05 ^Bb^	0.76 ± 0.04 ^A^	0.70 ± 0.04 ^ABa^	0.025	0.008
SR	0.84 ± 0.02 ^A^	0.66 ± 0.03 ^B^	0.81 ± 0.05 ^A^	0.67 ± 0.04 ^B^	0.021	0.001
Initial BSFL total weight (g WW)	2.90 ± 0.49	2.94 ± 0.5	2.91 ± 0.49	3.07 ± 0.51	0.512	0.996
BSFP weight ^1^ (g WW)	4.21 ± 0.39 ^ab^	3.51 ± 0.41^b^	4.90 ± 0.41^a^	4.11 ± 0.44 ^ab^	0.212	0.140
BSF weight ^1^ (g WW)	8.31 ± 0.34 ^A^	7.91 ± 0.43 ^ABa^	7.39 ± 0.19 ^ABab^	6.86 ± 0.32 ^Bb^	0.180	0.021
FCR_W_	13.13 ± 1.97	15.23 ± 3.14	14.43 ± 1.39	19.16 ± 3.08	1.263	0.370
FCR_D_	4.96 ± 0.76	5.89 ± 1.25	5.22 ± 0.54	7.11 ± 1.16	0.494	0.418

BSFLs = black soldier fly larvae; n = number of specimens; BSFPs = black soldier fly prepupae; total BSFs = count of all the BSF development stages at harvest; SR = survival rate (total BSFs to initial BSFLs ratio); WW = wet weight; FCR_W_ = feed conversion ratio based on the diet as fed; FCR_D_ = feed conversion ratio based on the dry matter of the diet; SEM = standard error for the mean; ^A,B^ values within a row with different superscripts are significantly different at *p* < 0.01; ^a,b^ values within a row with different superscripts are significantly different at *p* < 0.05. ^1^ Cumulate BSFPs (or BSF) weight per larvarium, as recorded at harvest. TMD1: high-nonfiber carbohydrate content total mixed diet; TMD2: high-fiber content total mixed diet; TMD3: high-protein content total mixed diet.

**Table 5:** Proximate composition (g/kg DM; unless otherwise stated) of black soldier fly prepupae (BSFPs) reared on the control diet (C) or experimental diets (TMD1–TMD3).

Proximate Composition	C	TMD1	TMD2	TMD3	SEM	*p*
DM (g/Kg WW)	333 ± 13	322 ± 7	344 ± 9	332 ± 7	4.7	0.443
ASH	101 ± 31	55 ± 19	110 ± 15	89 ± 22	11.0	0.358
CP	340 ± 10 ^A^	222 ± 18 ^B^	347 ± 18 ^A^	342 ± 11 ^A^	13.0	<0.001
EE	330 ± 6 ^B^	469 ± 18 ^A^	319 ± 15 ^B^	325 ± 17 ^B^	14.7	<0.001
CP_DEFATTED_	507 ± 14 ^A^	414 ± 19 ^B^	507 ± 17 ^A^	506 ± 5 ^A^	10.9	<0.001
CT	77 ± 8	96 ± 10	90 ± 5	93 ± 11	4.3	0.469
ROM	243 ± 11 ^a^	208 ± 10 ^b^	233 ± 9 ^ab^	232 ± 9 ^ab^	5.3	0.105

DM = dry matter; WW = wet weight; CP = crude protein; CP_DEFATTED_ = crude protein on defatted dry matter base; EE = ether extract; CT = chitin; ROM = residual organic matter calculated as [100 - (CP + EE + CT + Ash)]; SEM = standard error for the mean; ^A,B^ values within a row with different superscripts are significantly different at *p* < 0.01; ^a,b^ values within a row with different superscripts are significantly different at *p* < 0.05. TMD1: high-nonfiber carbohydrate content total mixed diet; TMD2: high-fiber content total mixed diet; TMD3: high-protein content total mixed diet.

**Table 6:** Yield (g/larvarium) of dry matter, crude protein, ether extract, and residual organic matter for BSFPs reared on the control (C) or experimental diets (TMD1–TMD3).

Nutrient Yields	C	TMD1	TMD2	TMD3	SEM	*p*
DMY	2.57 ± 0.36	3.08 ± 0.38	2.73 ± 0.3	2.77 ± 0.23	0.155	0.732
CPY	0.87 ± 0.12	0.68 ± 0.09	0.96 ± 0.14	0.95 ± 0.09	0.058	0.295
EEY	0.86 ± 0.13 ^B^	1.45 ± 0.19 ^A^	0.87 ± 0.1 ^B^	0.90 ± 0.08 ^B^	0.081	0.012
ROM	0.61 ± 0.07	0.65 ± 0.09	0.63 ± 0.06	0.63 ± 0.04	0.031	0.981

DMY = dry matter yield per larvarium; CPY = crude protein yield per larvarium; EEY = ether extract yield per larvarium; ROMY = residual organic matter yield per larvarium; SEM = standard error for the mean. ^A,B^ values within a row with different superscripts are significantly different at *p* < 0.01 TMD1: high-nonfiber carbohydrate content total mixed diet; TMD2: high-fiber content total mixed diet; TMD3: high-protein content total mixed diet.

**Table 7:** Total detected fatty acid (TDFA) content (mg/100 g DM) and main fatty acid content (g/100 g of TDFA) of black soldier fly prepupae (BSFPs) reared on the control (C) or the experimental diets (TMD1–TMD3).

Fatty Acids	C	TMD1	TMD2	TMD3	SEM	*p*
TDFA	30.82 ± 2.56 ^B^	44.02 ± 4.82 A	29.72 ± 3.27 ^B^	30.4 ± 4.69 ^B^	1.45	<0.001
C10:0	0.82 ± 0.11	0.81 ± 0.05	0.85 ± 0.06	1.02 ± 0.04	0.038	0.178
C12:0	54.31 ± 2.35 ^B^	62.75 ± 0.89 ^A^	55.22 ± 1.75 ^B^	55.78 ± 0.95 ^B^	1.024	0.005
C12:1	0.05 ± 0.01 ^a^	0.03 ± 0.01 ^b^	0.04 ± 0.00 ^ab^	0.05 ± 0.00 ^a^	0.003	0.049
C14:0	8.77 ± 0.11 ^B^	10.70 ± 0.37 ^A^	9.30 ± 0.22 ^B^	8.84 ± 0.10 ^B^	0.194	<0.001
C14:1 *cis* + C15:0	0.49 ± 0.08	0.38 ± 0.02	0.46 ± 0.02	0.48 ± 0.01	0.021	0.235
C16:0	12.04 ± 0.75	10.87 ± 0.46	11.94 ± 0.60	11.95 ± 0.41	0.285	0.441
C16:1	2.47 ± 0.32	2.78 ± 0.10	2.4 ± 0.12	2.43 ± 0.08	0.092	0.463
C18:0	1.18 ± 0.11 ^a^	0.92 ± 0.08 ^b^	1.09 ± 0.07 ^ab^	1.09 ± 0.06 ^ab^	0.044	0.207
C18:1 *cis* 9	6.4 ± 0.80 ^a^	4.75 ± 0.21 ^b^	5.3 ± 0.34 ^ab^	5.08 ± 0.18 ^ab^	0.250	0.093
C18:1 *cis* 11	0.34 ± 0.02 ^A^	0.16 ± 0.02 ^B^	0.43 ± 0.05 ^A^	0.42 ± 0.06 ^A^	0.029	0.001
C18:2 n-6	8.07 ± 1.25 ^A^	3.69 ± 0.11 ^B^	8.5 ± 1.11 ^A^	7.65 ± 0.77 ^A^	0.587	0.005
C18:3 n-6	0.02 ± 0.00 ^A^	0.01 ± 0.00 ^B^	0.02 ± 0.00 ^A^	0.01 ± 0.00 ^A^	0.001	0.007
C18:3 n-3	0.54 ± 0.06 ^ABa^	0.37 ± 0.01 ^Bb^	0.64 ± 0.07 ^A^	0.67 ± 0.03 ^A^	0.034	0.003
C20:0	0.03 ± 0.00 ^A^	0.02 ± 0.00 ^B^	0.03 ± 0.00 ^A^	0.03 ± 0.00 ^A^	0.002	0.002
C20:1 *cis* 11	0.02 ± 0.00 ^A^	0.01 ± 0.00 ^B^	0.02 ± 0.00 ^A^	0.02 ± 0.00 ^A^	0.001	<0.001
C20:2 n-6	0.01 ± 0.00	0.01 ± 0.00	0.00 ± 0.00	0.01 ± 0.01	0.002	0.659
C22:0	0.07 ± 0.02	0.07 ± 0.04	0.05 ± 0.03	0.05 ± 0.02	0.018	0.598
Unidentified FA	1.04 ± 0.23	1.28 ± 0.54	0.60 ± 0.27	0.83 ± 0.36	0.225	0.532
ΣSFA	79.58 ± 1.55 ^B^	86.89 ± 0.57 ^A^	81.05 ± 1.15 ^B^	81.35 ± 0.35 ^B^	0.750	<0.001
Σiso + anteiso FA	0.47 ± 0.09 ^B^	0.77 ± 0.30 ^A^	0.39 ± 0.14 ^B^	0.34 ± 0.09 ^B^	0.201	<0.001
ΣMUFA	10.55 ± 0.9 ^a^	8.49 ± 0.39 ^b^	9.01 ± 0.42 ^a^	8.87 ± 0.16 ^a^	0.300	0.066
ΣMUFA *trans*	8.77 ± 0.11	10.7 ± 0.37	9.3 ± 0.22	8.84 ± 0.1	0.090	0.301
ΣPUFA	9.87 ± 0.9 ^A^	4.62 ± 0.27 ^B^	9.94 ± 0.86 ^A^	9.78 ± 0.27 ^A^	0.563	<0.001
ΣUFA/ΣSFA	0.26 ± 0.02 ^A^	0.15 ± 0.01 ^B^	0.24 ± 0.02 ^A^	0.23 ± 0.01 ^A^	0.011	0.001
ΣMUFA/ΣSFA	0.13 ± 0.01 ^A^	0.1 ± 0.01 ^B^	0.11 ± 0.01 ^A^	0.11 ± 0 ^A^	0.005	0.032
ΣPUFA/ΣSFA	0.13 ± 0.01 ^A^	0.05 ± 0 ^B^	0.12 ± 0.01 ^A^	0.12 ± 0 ^A^	0.008	<0.001
ΣPUFA/ΣMUFA	0.96 ± 0.08 ^A^	0.55 ± 0.03 ^B^	1.1 ± 0.08 ^A^	1.1 ± 0.03 ^A^	0.055	<0.001

SFA = saturated fatty acids; MUFA = monounsaturated fatty acids; PUFA = polyunsaturated fatty acids; UFA = unsaturated fatty acids; SEM = standard error for the mean ^A,B^ values within a row with different superscripts are significantly different at *p* < 0.01; ^a,b^ values within a row with different superscripts are significantly different at *p* < 0.05. TMD1: high-nonfiber carbohydrate content total mixed diet; TMD2: high-fiber content total mixed diet; TMD3: high-protein content total mixed diet.

**Table 8:** Apparent bioconcentration factors (aBCFs) for individual fatty acids found in black soldier fly prepupae (BSFPs) reared on the control (C) or experimental diets (TMD1–TMD3).

aBCFs	C	TMD1	TMD2	TMD3	SEM	*p*
aBCF_C12:0_	84.86 ± 3.68 ^D^	169.6 ± 2.41 ^C^	250.98 ± 7.94 ^B^	328.09 ± 5.58 ^A^	19.075	<0.001
aBCF_C14:0_	17.54 ± 0.23 ^B^	17.84 ± 0.61 ^AB^	15.76 ± 0.38 ^C^	22.1 ± 0.25 ^A^	0.519	<0.001
aBCF_C16:0_	0.49 ± 0.03 ^a^	0.34 ± 0.01 ^b^	0.43 ± 0.02 ^ab^	0.44 ± 0.02 ^ab^	0.015	0.001
aBCF_C16:1_	7.49 ± 0.98 ^B^	13.89 ± 0.50 ^A^	8.00 ± 0.40 ^B^	8.11 ± 0.28 ^B^	0.614	<0.001
aBCF_C18:0_	0.78 ± 0.08 ^ABb^	0.58 ± 0.05 ^Bc^	0.98 ± 0.07 ^Aa^	1.00 ± 0.06 ^A^	0.046	<0.001
aBCF_C18:1 *cis* 9_	0.31 ± 0.04	0.36 ± 0.02	0.38 ± 0.02	0.38 ± 0.01	0.013	0.211
aBCF_C18:1 *cis* 11_	0.48 ± 0.03 ^a^	0.3 ± 0.04 ^b^	0.44 ± 0.05 ^a^	0.43 ± 0.06 ^ab^	0.026	0.061
aBCF_C18:2 N-6_	0.17 ± 0.03 ^A^	0.08 ± 0.01 ^Bb^	0.17 ± 0.02 ^A^	0.15 ± 0.02 ^ABa^	0.012	0.008
aBCF_C18:3 N-3_	0.13 ± 0.01^ABa^	0.09 ± 0.00 ^Bb^	0.15 ± 0.02 ^A^	0.13 ± 0.01 ^ABb^	0.007	0.015
aBCF_C20:1 *cis* 11_	n.c.	0.06 ± 0.01 ^B^	0.08 ± 0.01 ^A^	0.07 ± 0.01 ^AB^	0.004	0.005

SEM = standard error for the mean; n.c. = not calculable; ^A,B,C,D^ values within a row with different superscripts are significantly different at *p* < 0.01; ^a,b,c^ values within a row with different superscripts are significantly different at *p* < 0.05. TMD1: high-nonfiber carbohydrate content total mixed diet; TMD2: high-fiber content total mixed diet; TMD3: high-protein content total mixed diet.

**Table 9:** Correlations among the fatty acid concentrations in black soldier fly prepupae (BSFPs) and the fatty acid concentrations in their diets.

FA	r	FA	r
C12:0	−0.06	C18:2 n-6	0.41 *
C14:0	0.59 **	C18:3 n-6	0.41 *
C16:0	−0.33 *	C18:3 n-3	0.45 *
C16:1	−0.31	C20:0	0.25
C18:0	−0.16	C20:1 *cis* 11	0.52 *
C18:1 *cis* 9	0.50 *	Σiso + anteiso FA	−0.42 *
C18:1 *cis* 11	0.72 ***	ΣMUFA *trans*	−0.25

FA = fatty acid or FA group; r = Person’s product moment correlation coefficient; ^*^ *p* < 0.05; ^**^ *p* < 0.01; ^***^ *p* < 0.001.

## Supplementary Materials

The following are available online at https://www.mdpi.com/2076-2615/9/4/178/s1. Table S1: Effect of decolorization treatment with potassium permanganate (KMnO_4_) on the weight loss of three different samples of black soldier fly prepupae (BSFPs) meal submitted to the demineralization/deproteinization procedure that was described by Liu et al. [36].
