# Supplementary material for: The Effects of Diet Formulation on the Yield, Proximate Composition, and Fatty Acid Profile of the Black Soldier Fly (Hermetia illucens L.) Prepupae Intended for Animal Feed
Source: Animals (Basel). 2019 Apr 19;9(4):178. doi: 10.3390/ani9040178 (PMC6523828; doi:10.3390/ani9040178)
Supplement: Supplementary file 1 [file animals-09-00178-s001.pdf]

animals

Effects of Diet Formulation on the Yield, Proximate Composition and Fatty Acid Profile of the Black Soldier Fly (*Hermetia illucens* L.) Prepupae Intended for Animal Feed.

Pier Paolo Danieli[1]. Carola Lusiana[2]. Laura Gasco[2]. Andrea Amici. Bruno Ronchi[1]

[1] Department of Agriculture and Forest Sciences; University of Tuscia, Viterbo, Italy; amici@unitus.it; ronchi@unitus.it
[2] Department of Agricultural Forest and Food Sciences; University of Torino, Grugliasco (TO), Italy; carola.lussiana@unito.it; laura.gasco@unito.it
* Correspondence: danieli@unitus.it; Tel.: +39-0761-357349

Table S1. Effect of decolorization treatment with potassium permanganate ($KMnO4$) on the weight loss of three different samples of black soldier fly prepupae (BSFP) meal submitted to the demineralization/deproteinization procedure described by Liu et al. [36].

Sample	Total initial weight [1] (g)	Total post demineralization/deproteinization treatment weight (g)	Total post-decolorization treatment weight (g)
#1	0.095	0.046	0.046
#2	0.094	0.045	0.045
#3	0.099	0.049	0.049
Average	0.096	0.047	0.047

[1] Total weight includes the weight of the ANKOM modified bag and the weight of BSFP meal.
